# Effects of dietary tryptophan and phenylalanine–tyrosine depletion on phasic alertness in healthy adults – A pilot study

**DOI:** 10.3402/fnr.v59.26407

**Published:** 2015-04-29

**Authors:** Patricia Hildebrand, Werner Königschulte, Tilman Jakob Gaber, Sarah Bubenzer-Busch, Katrin Helmbold, Caroline Sarah Biskup, Karl-Josef Langen, Gereon Rudolf Fink, Florian Daniel Zepf

**Affiliations:** 1 Clinic for Child and Adolescent Psychiatry, Psychosomatics and Psychotherapy, JARA Brain, Medical Faculty, RWTH Aachen University, Aachen, Germany; 2 Institute of Neuroscience and Medicine (INM-3, -4, -5), Research Centre Jülich, Jülich, Germany; 3 Department of Nuclear Medicine, RWTH Aachen University Hospital, Aachen, Germany; 4 Department of Neurology, University of Cologne, Cologne, Germany; 5 Department of Child and Adolescent Psychiatry, School of Psychiatry and Clinical Neurosciences & School of Paediatrics and Child Health, Faculty of Medicine, Dentistry and Health Sciences, The University of Western Australia, Perth, Australia; 6 Specialised Child and Adolescent Mental Health Services (CAHMS), Department of Health in Western Australia, Perth, WA, Australia

**Keywords:** dietary challenge procedures, amino acids, acute tryptophan depletion, phenylalanine–tyrosine depletion, serotonin, dopamine, phasic alertness

## Abstract

**Background:**

The synthesis of the neurotransmitters serotonin (5-HT) and dopamine (DA) in the brain can be directly altered by dietary manipulation of their relevant precursor amino acids (AA). There is evidence that altered serotonergic and dopaminergic neurotransmission are both associated with impaired attentional control. Specifically, phasic alertness is one specific aspect of attention that has been linked to changes in 5-HT and DA availability in different neurocircuitries related to attentional processes. The present study investigated the impact of short-term reductions in central nervous system 5-HT and DA synthesis, which was achieved by dietary depletion of the relevant precursor AA, on phasic alertness in healthy adult volunteers; body weight–adapted dietary tryptophan and phenylalanine–tyrosine depletion (PTD) techniques were used.

**Methods:**

The study employed a double-blind between-subject design. Fifty healthy male and female subjects were allocated to three groups in a randomized and counterbalanced manner and received three different dietary challenge conditions: acute tryptophan depletion (ATD, for the depletion of 5-HT; *N*=16), PTD (for the depletion of DA; *N*=17), and a balanced AA load (BAL; *N*=17), which served as a control condition. Three hours after challenge intake (ATD/PTD/BAL), phasic alertness was assessed using a standardized test battery for attentional performance (TAP). Blood samples for AA level analyses were obtained at baseline and 360 min after the challenge intake.

**Results:**

Overall, there were no significant differences in phasic alertness for the different challenge conditions. Regarding PTD administration, a positive correlation between the reaction times and the DA-related depletion magnitude was detected via the lower plasma tyrosine levels and the slow reaction times of the first run of the task. In contrast, higher tryptophan concentrations were associated with slower reaction times in the fourth run of the task in the same challenge group.

**Conclusion:**

The present study is the first to demonstrate preliminary data that support an association between decreased central nervous system DA synthesis, which was achieved by dietary depletion strategies, and slower reaction times in specific runs of a task designed to assess phasic alertness in healthy adult volunteers; these findings are consistent with previous evidence that links phasic alertness with dopaminergic neurotransmission. A lack of significant differences between the three groups could be due to compensatory mechanisms and the limited sample size, as well as the dietary challenge procedures administered to healthy participants and the strict exclusion criteria used. The potential underlying neurochemical processes related to phasic alertness should be the subject of further investigations.

Attention is one of the most widely studied topics within the experimental and clinical neurosciences. Regarding the underlying neurobiological and neurochemical properties, attentional processes have been linked to changes in central nervous dopaminergic (DA) and serotonergic (5-HT) neurotransmission. Of note, the central nervous system availability of these particular neurotransmitters can be manipulated by changing the intake of their respective precursor amino acids (AA) using dietary depletion strategies (1–12); this approach enables short-term manipulations of these neurotransmitters within experimental settings. As a consequence, the assessment of attentional performance after dietary depletion of central nervous system DA and 5-HT synthesis enables conclusions to be drawn regarding the impact of these neurotransmitters on different aspects of attention.

Current evidence suggests that the attention system in humans consists of various anatomical networks, which are specified into different functions (13), and must be differentiated from processing systems, which are responsible for memory storage, decision-making and problem-solving activities (14). Posner and Petersen (15) described three different networks that comprised the attention system: orienting, executive, and alerting. The main task of the orienting network is to prioritize all sensory inputs. When processing information, a selection for a modality or a location occurs. The executive network is primarily involved in target detection. Different targets can be monitored at the same time; however, the moment of detection causes interferences, which lead to a slower detection of other stimuli (16). Alertness, which is the subject of the present study, can be subdivided into two domains, tonic and phasic. Tonic alertness is described as a particular form of vigilance. In contrast, phasic alertness is required to be prepared for an expected stimulus. The performance is better if a warning signal, such as a cued stimulus, is provided, depending on the interval between the warning signal and signal detection (17).

The anatomical correlates of alertness include a variety of different brain regions. The dorso-lateral prefrontal cortex (DLPFC) exhibits activation during phasic alertness tasks that are characterized by the use of a warning signal (18). Another region, which is of particular importance in the context of alertness and attention, is the anterior cingulate cortex (ACC) (19); the ACC is rich in dopaminergic projections and plays a critical role in attentional processes. Research has identified ACC dysfunctions in attention deficit hyperactivity disorder (ADHD), which is a neuropsychiatric disorder characterized by inattention, impulsiveness, and hyperactivity (20). PET and fMRI studies have identified activations in the frontal, parietal, thalamic, and brain-stem networks and have primarily demonstrated the right hemisphere to be involved in phasic alertness tasks (21).

The normal function of the alertness network is influenced by many neurotransmitters with complex interactions, including transport mechanisms and degradation pathways. The central nervous system availability of neurotransmitters that are relevant for attentional processes (i.e. alertness) include, among others, 5-HT, DA, and noradrenaline (NE) (4). These particular neurotransmitter systems can be modulated through a variety of different mechanisms: polymorphisms in monoamine oxidase A (MAO-A), one of the most important enzymes in the brain, and the degradation of biogenic amines (including the key neurotransmitters 5-HT, DA, and NE) significantly influence reaction times in the attention network test (22). The same effect applies for catechol-*O*-methyl-transferase COMT, an enzyme that inactivates catecholaminergic neurotransmitters and impacts the dopaminergic signal-to-noise ratio (SNR). The SNR refers to local cortical microcircuits which are essential to cortical representation of external and internal stimuli (23). Moderate DA elevations thereby improve signaling to a certain extent (24). Furthermore, the perception of external stimuli is subject to top-down control associated with the prefrontal cortex, which receives ascending information from the major neurotransmitter systems, such as DA, 5-HT, acetylcholine (Ach), and noradrenaline (NA) (25).

The efficiency of the alerting network is primarily manipulated by NE, whereas the alerting and orienting networks are thought to be related to cholinergic and DA-related modulations, with evidence that originates from research involving pharmacological manipulations (26). Research on neuropharmacological mechanisms often uses the strategy of changing the central nervous availability of neurotransmitters by manipulating the availability of their relevant precursor AAs, which, in turn, can be changed by established dietary depletion strategies using established procedures (2–12, 27–29) .

In general, plasma concentrations of AAs are not only regulated by the amount of food ingested but also by the composition of macronutrients that determine the ratio of AAs to each other in plasma, and thus can affect their availability in the brain through a variety of mechanisms. Because the underlying enzymes for neurotransmitter synthesis are not fully saturated, changes in the concentrations of the relevant precursor AAs are one possible mechanism to impact neurotransmitter synthesis. Changes in the ratio of carbohydrates to proteins ingested can cause differences in plasma tryptophan and LNAAs ratios. Meals rich in proteins cause a decrease in the ratio of plasma tryptophan to LNAAs, in particular as they provide less TRP than LNAAs and thus facilitate influx of AAs other than TRP into the brain.

In contrast, insulin secretion after a carbohydrate-rich meal will likely increase the ratio of plasma TRP to LNAAs via an increased AA uptake into the liver and muscles. In the central nervous system, insulin is involved in weight control and regulation through the reduction of energy intake (26). Those two mechanisms (insulin secretion after carbohydrate intake and AA metabolism in the liver) are almost balanced if carbohydrates and proteins are ingested in a ratio of 5:6 (28). The same applies to changes in tyrosine concentrations. However, it needs to be mentioned that the effects of tyrosine on central nervous dopamine synthesis are thought to be less pronounced than the effects of TRP for brain 5-HT synthesis, the reason for this being related to the activity of the enzyme tyrosine hydroxylase whose catalytic activity does not only depend on enzyme saturation but also on the availability of its cofactor tetrahydrobiopterin (29). Finally, disorders like premenstrual syndrome, seasonal depression and affective disorders are characterized by disturbances in mood and appetite, possibly associated with craving for carbohydrates. Moreover, research suggests that carbohydrates consumed can increase 5-HT availability in the brain and can thus impact mood (30).

The present work focuses on phasic alertness and its neurochemical underpinnings modulated by the neurotransmitters 5-HT and DA and the dietary availability of their respective precursor AAs. Tonic and phasic alertness, which are the two main components of the alertness construct, are closely related to arousal in humans. As previously discussed, these neurotransmitter systems are thought to be related to the attention system in the human brain. The present study investigates the effects of diminished central nervous system 5-HT and DA synthesis, which was achieved by dietary acute tryptophan depletion (ATD, which decreases central nervous system 5-HT synthesis) and phenylalanine–tyrosine depletion (PTD, which decreases DA synthesis in the brain), on phasic alertness in healthy adult volunteers. This research is of particular importance to gain further understanding of the underlying mechanisms related to attentional processes and to develop novel strategies for interventions in patients with attentional problems, which potentially use neurodietary concepts and related approaches. Although the results of studies that have focused on the effects of ATD on memory, attention, and executive functions are somewhat inconsistent, we expected that reduced serotonergic inhibition on monoaminergic and cholinergic systems would improve attentional control (27, 30). Deficiencies in attention, memory, and motivational and behavioral processes have been identified in patients with ADHD, a neuropsychiatric disorder with an etiology most likely associated with changes in dopaminergic turnover. The most common treatment for ADHD is methylphenidate, which increases extracellular DA in the brain and thereby reduces impulsiveness and inattentiveness. A correlation between DA and alterations in attentional processes has been demonstrated (31); however, mixed findings have been reported in studies that used PTD or systematic administration of selective DA receptor agonists and antagonists. Therefore, we expected that PTD would lead to impaired attentional control and subsequently slower reaction times in a phasic alertness task.

## Methods

### Study sample

In the present study, data from a sample that comprised 50 healthy adult subjects were analyzed. The mean age was 24.7±4.6 years. Gender within the study sample was balanced (25 females, 25 males) and almost equally distributed over the three subgroups with respect to the depletion of 5-HT (ATD; *N*=16) and DA (PTD; *N*=17) and the administration of a balanced amino acid load (BAL; *N*=17), which served as a control condition. The characteristics of the study sample are shown in Table 1. The study was assessed and approved by the local ethics committee and was conducted in accordance with the Helsinki Declaration. Each subject was provided with a complete description of the study, and written and verbal informed consents were obtained prior to participation. The participants were financially compensated for their participation.

**Table 1:** The characteristics (age in years, weight in kg) of the different study groups with regard to their distribution over three groups that received acute tryptophan depletion (ATD, to decrease central nervous system serotonin synthesis), phenylalanine–tyrosine depletion (PTD, to decrease central nervous system dopamine synthesis), or a balanced amino acid load (BAL, which served as a control condition)

	Age (years±SD)	Weight (kg±SD)
ATD	24.38±4.13	71.22±13.54
PTD	23.59±3.12	70.41±11.65
BAL	26.24±5.86	74.68±17.86

Data are presented as the mean±standard deviation.

Only healthy adults without acute or previous psychiatric or acute somatic disorders were included (ineligible individuals were excluded prior to participation via interview and individual history). A screening procedure for mental disorders (SCID; Structured Clinical Interview for DSM-IV axis I disorders) was implemented to exclude DSM-IV Axis I disorders known to be associated with changes in neurotransmitter systems of the human brain. This study was part of a larger testing battery that included an fMRI-experiment on social decision-making, which is the subject of a different publication. In addition to the standard contraindications of an MRI examination, an IQ below 85, substance abuse and pregnancy were exclusion criteria. Moreover, the individual BMI of every subject was calculated as surrogate for possible pathological differences in the metabolism of AA. All subjects came from the same catchment area, and most of the participants were students of similar educational and socio-economic background. In this way, the study sample kept as homogenous as possible to avoid further factors influencing behavioral and biological testing results.

A body weight–adapted dietary depletion procedure termed Moja-De was used (32). One major advantage of the body weight–adapted dietary depletion procedure is its improved tolerability, which also enables its use in children and adolescents (3, 29, 33, 34). In addition, the Moja-De procedure also considers baseline plasma TRP concentrations because of a positive correlation between baseline TRP and body weight (2). Moreover, the ATD challenge procedure has recently been validated in an animal model (1) and demonstrated a significant decrease in TRP influx into the brain following administration in healthy adults (3). In addition, a recent study demonstrated that a PTD challenge administration in accordance with body weight affected DA synthesis and reduced TYR and HVA concentrations in the brain (35).

### Study design

A randomized, double-blind, between-subject design was used (the reason for this was that some of the behavioral testing batteries administered to the subjects are difficult to be used in a repeated measures design because of learning effects; these data are subject to different publications). The subjects were assigned to three groups in a randomized and counterbalanced manner: dietary depletion of 5-HT (ATD), depletion of DA (PTD), and a control group (BAL). Testing was conducted on one study day for each subject. The present publication focuses on the behavioral data related to aspects of phasic alertness. AA administration and the assessment of attentional performance as indexed by phasic alertness were followed by an fMRI experiment, which examined aspects of social decision-making performed 300 min after AA administration (the data from this experiment are the subject of a different publication).

### Depletion procedure

ATD is a neurodietary method used to achieve a short-term reduction in central nervous system 5-HT synthesis, which induces short-term serotonergic dysfunction. This method enables the examination of the underlying effects of 5-HT on different behavioral, affective and biological parameters. 5-HT, which cannot cross the blood–brain barrier, must be synthesized in the brain. The essential AA tryptophan (TRP) serves as the biological precursor of 5-HT, and TRP must be obtained with daily nutrition intake. TRP uptake into the brain across the blood–brain barrier is mediated by a transport protein called L-1, which facilitates AA influx across the blood–brain barrier. In addition to TRP, L-1 is also utilized by tyrosine ([TYR], which serves as a precursor for DA) and other large neutral amino acids (LNAAs). As a consequence, changes in the concentrations of AAs that use L-1 can impact AA influx and biological precursor availability for neurotransmitters in the brain. The rate-limiting step in central nervous system 5-HT synthesis is the hydroxylation of TRP into 5-hydroxytryptophan, which is controlled by the enzyme tryptophan hydroxylase 2 (TPH-2). Under physiological conditions, this particular enzyme is only saturated up to 50%. Thus, dietary changes in physiological 5-HT-related precursor availability (in terms of TRP) automatically lead to altered enzyme saturations, which can then lead to changes in the synthesis rate (36). In ATD, participants are provided a TRP-free mixture of LNAAs for oral intake. The resulting diminished TRP influx into the brain is attributed to combined effects: First, the administered AAs compete with TRP for uptake into the brain. Second, a stimulated protein biosynthesis in the liver, where endogenous TRP is consumed and removes TRP from plasma stores, must also be considered. Moreover, a passive diffusion of TRP across the blood–brain barrier contributes to the overall TRP balance in the central nervous system. PTD is based on the same underlying principle as ATD. The corresponding AA mixtures used for PTD are lacking in the catecholamine precursor phenylalanine (PHE). Although PHE belongs to the group of essential AAs, it is the lack of TYR (a dispensable or nonessential AA) that reduces DA synthesis because of reduced substrate availability for the key regulatory enzyme in catecholamine synthesis, tyrosine hydroxylase, which is normally not fully saturated (37). As previously discussed, both DA precursors use the same transport system as TRP to cross the blood–brain barrier. The saturable L-1 transporter mediates a competitive, electroneutral facilitated diffusion of the neutral AAs, which depends on their affinities to the transporter and their blood concentrations (38).

In the present study, ATD-, PTD-, and BAL-related AA mixtures were provided and administered. The AA mixtures were stored in brown wide mouth light absorbing bottles at 8°C until use. The quantities of the different AA mixtures for the three different conditions (ATD, PTD, and BAL) are shown in Table 2.

**Table 2:** The amino acid quantities (amount per 10 kg body weight) for the challenge conditions, including acute tryptophan depletion (ATD, to decrease central nervous system serotonin synthesis), phenylalanine–tyrosine depletion (PTD, to decrease central nervous system dopamine synthesis), and a balanced amino acid load (BAL, which serves as a control condition)

Amino acids	ATD (g)	PTD (g)	BAL (g)
L-Phenylalanine	1.32	–	1.32
L-Leucine	1.32	1.32	1.32
L-Isoleucine	0.84	0.84	0.84
L-Methionine	0.5	0.5	0.5
L-Valine	0.96	0.96	0.96
L-Threonine	0.6	0.6	0.6
L-Lysine-HCl (=L-Lysine 0.9 g/10 kg)	1.2	1.2	1.2
L-Tryptophan	–	0.7	0.7

All participants were subjected to an overnight fast after 8 pm the day prior to study participation. The participants were allowed to drink water ad libitum throughout the respective study day. Prior to administration, the AAs of the respective challenge conditions were dissolved in water using a milk frother. Before the AA beverages were administered, the body weight was verified, as well as a drug screen; a pregnancy test was administered to female subjects. The first baseline blood sample was collected prior to the ingestion of the respective challenge beverage (T1); the second blood sample was collected approximately 360 minutes after the challenge intake. The participants were instructed to ingest the entire beverage in one go, if possible. Following the oral ingestion of the corresponding AA mixture dissolved within a beverage, the participants received a standardized, low-protein breakfast.

Subjective mood was assessed on the basis of a questionnaire (39) at seven different time points throughout the study day; the assessments began prior to the first blood sample at baseline, followed by assessments at hourly intervals and after the MRI scan (T2). The participants were provided their own room for the entire study day.

### Mood assessment

The ASTS (39), a German version of the ‘Profile of mood states’ (POMS), is a self-assessment questionnaire that aims to assess different aspects of mood and subjective well-being. The areas assessed included grief, despair, fatigue, positive mood, and anger. The ASTS is a reliable tool for the subjective assessment of current mood (39). The mood data are the subject of a different publication.

### Assessment of phasic alertness

Three hours after the ingestion of the AA mixtures, attentional performance was assessed using a neuropsychological test battery for attentional performance (40). This publication focuses on phasic alertness and the potential changes in this parameter with regard to the administration of ATD, PTD and BAL, other aspects of attentional performance are subject to different publications. This time point represents the time with sufficient depletion, which has been demonstrated by research in humans (3) and rodents (1). To ensure absolute silence during the assessment of attentional performance, testing was conducted in the participant's room without interruption. The overall testing time for the assessment of phasic alertness was approximately 20 min per participant. A short training session prior to the final assessment was administered for each participant. The computerized measurements of attentional performance involved the positioning of the subjects in a comfortable way. The subjects were instructed to maintain an approximately one meter distance between them and the PC screen and to leave their hand in a calm manner on the response key. No feedback regarding individual performance was provided. The testing conditions were similarly maintained for all participants.

The task included four blocks, with each consisted of 20 target stimuli, and the reaction times to a target stimulus were assessed. Phasic alertness was measured by the reaction times to a target stimulus preceded by a signal tone, which followed an ABBA design (A: trial without a preceding warning signal, B: trial with a preceding warning signal) and is a standardized procedure (40). The participants were instructed to react via button-press as quickly as possible to a cross (‘x’) that appeared at randomly varying intervals on a display screen. The task measures included the median reaction time, standard deviation of the reaction time, omission errors (misses), and commission errors (false alarms).

### Blood samples

To determine the individual concentrations of TRP, TYR, and PHE, several blood samples were obtained at different time points. One blood sample was obtained under baseline conditions, and an additional blood sample (T2) was obtained after the MRI experiment (approximately 360 min after the challenge intake) to quantify the amount of depletion after the challenge (ATD/PTD) or control (BAL) administration.

All blood samples were obtained and treated as follows: after 30 min of clotting time, the blood was centrifuged at 2,500 rtb for 10 min, pipetted into crya vials, and retained in a freezer at −80°C until final analysis. All samples were measured and evaluated for the concentrations of TRP, PHE, TYR and competing branched chained amino acids (BCAAs). The method for the measurement of TRP, PHE, and TYR concentrations was an immunosorbent assay (ELISA) kit (Immundiagnostik AG, Bensheim, Germany), which was used in accordance with the manufacturer's instructions. The BCAA concentrations were determined using a commercially available enzyme BCAA test kit (Immundiagnostik AG, Bensheim, Germany).

### Statistical analysis

Statistical evaluation of the data was performed using SPSS 20 software (IBM Statistics). The potential group differences were analyzed using Kruskal–Wallis tests with ‘challenge administration’ as a between-group factor and reaction times for each run of the task as a dependent variable. To study the impact of dietary depletion strategies on reaction times, the differences in concentrations for each relevant AA before and after challenge administration in relation to the differences in the competing AAs before and after depletion were calculated as an index of the depletion magnitude [TRP ratio]: (TRP_T1_/BCAA_T1_+PHE_T1_+TYR_T1_)-(TRP_T2_/BCAA_T2_+PHE_T2_+TYR_T2_); TYR ratio: (TYR_T1_/BCAA_T1_+PHE_T1_+TRP_T1_)-(TYR_T2_/BCAA_T2_+PHE_T2_+TRP_T2_); PHE ratio: (PHE_T1_/BCAA_T1_+TRP_T1_+TYR_T1_)-(PHE_T2_/BCAA_T2_+TRP_T2_+TYR_T2_). Using a group-wise dimensional approach, a Spearman rho correlation was conducted for each group to identify significant correlations between the depletion magnitude and median reaction times (RT_mdn_) of the phasic alertness task.

In addition to the RT_mdn_, the alertness reaction, which indicates increasing levels of attention, was assessed using *t*-values for PA that were assessed within the previously described testing battery; it was defined as the subject's ability to shorten their own reaction times in trials in which the relevant stimulus was preceded by a signal tone (runs two and three). The level of statistical significance was set at *p*<0.05. Because of the explorative nature of this study and to not overlook significant relationships, this study did not incorporate alpha-adjustment. As an estimate of the relevant effect sizes, Cohen's *d* values were calculated. This study was conducted as a pilot in order to generate first data that allows for the calculation of sample sizes for future large-scale research. As for pilot studies power calculations are not an essential criterion, a post-hoc power analysis was conducted using StatMate (GraphPad, La Jolla, USA) for the calculation of sample sizes of such future studies.

## Results

### Neurochemical effects of the challenge administration

**Fig. 1:**
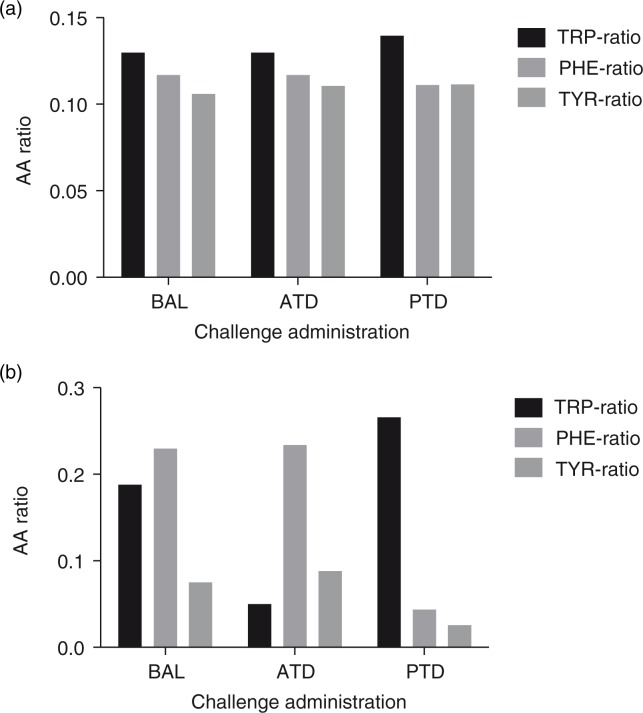
(a) The respective AA ratios prior to challenge administration. (b) The respective AA ratios after challenge administration.

In the assessment of the TRP-, PHE-, and TYR-ratios at T2, a Kruskal–Wallis test identified a significant effect for the factor challenge administration (TRP ratio: *p*=0.000, *χ* ^2^=36.14, *df*=2, 47; PHE ratio: *p*=0.000, *χ* ^2^=33.13, *df=*2, 47; and TYR ratio: *p*=0.000, *χ* ^2^= 34.26, *df*=2, 47). In particular, for T2 after ATD administration, a Wilcoxon–Mann–Whitney test demonstrated that the TRP ratio (*p*=0.000, U=0, *T=*136), PHE ratio (*p*=0.589, U=121, *T=*274), and TYR ratio (*p*=0.031, U=76, *T*=229) were significantly different than for BAL administration. With regard to the PTD challenge, similar results were identified with respect to the TRP ratio (*p*=0.000, U=58, *T*=211), PHE ratio (*p*=0.000, U=0, *T*=153), and TYR ratio (*p*=0.000, U=4, *T*=157) compared with BAL administration. Overall, these findings demonstrate that the depletion strategies used with regard to 5-HT (ATD) and DA (PTD) worked as intended. Moreover, there were no significant differences between the ATD, PTD, and BAL groups at baseline regarding the TRP ratio (*p*=0.29, *χ* ^2^=2.46, *df*=2, 47), PHE ratio (*p*=0.59, *χ* ^2^=1.07, *df*=2, 47), or TYR ratio (*p*=0.79, *χ* ^2^=0.47, *df*=2, 47).An overview of these findings is provided in Fig. 1a and b.

### Effects of the challenge administration on phasic alertness

The median of the reaction time (RT_mdn_) is a measure of general slowdown, whereas the standard deviation of the reaction time is indicative of fluctuations in attentional performance.

No significant differences regarding the *t*-values for phasic alertness or RT_mdn_ for the different dietary challenge conditions (ATD/PTD/BAL) were identified (Table 3).

**Table 3:** The values for the mean reaction times and the statistics (Kruskal–Wallis test) for each run of the task, the trials with and without a signal tone, and the *t-*values of phasic alertness

	Kruskal – Wallis test					

	*df*	*p-*value	Chi-squared values	Mean – ATD	Mean – PTD	Mean – BAL
*t*-values for phasic alertness	2, 47	0.8	0.73	0.03±0.07	0.01±0.05	0.04±0.08
Reaction times run 1	2, 47	0.4	1.84	240.69±28.23	228.06±25.84	234.41±33.61
Reaction times run 2	2, 47	0.77	0.52	234.56±26.03	230.76±25.74	229.29±25.13
Reaction times run 3	2, 47	0.39	1.88	238.38±27.46	228.41±22.97	224.88±22.1
Reaction times run 4	2, 47	0.49	1.43	249.06±34.36	236.12±30.65	240.29±27.9
Reaction times without a signal tone	2, 47	0.39	1.86	245.63±30.02	232.35±26.74	237.35±27.47
Reaction times with a signal tone	2, 47	0.57	1.12	236.06±25.35	229.29±22.56	226.82±20.8

### Effects of depletion magnitude on RT_mdn_

**Fig. 2:**
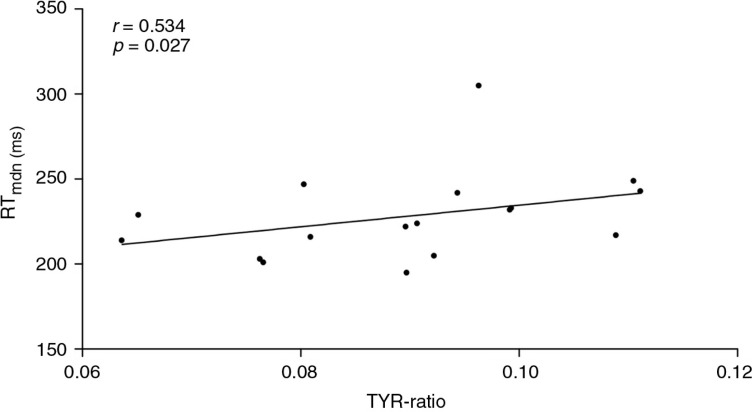
Correlation between plasma TYR-ratio and the median reaction time (milliseconds) in the first run of the task in the PTD-challenge group (*p*=0.027, *r*=0.534).

**Fig. 3:**
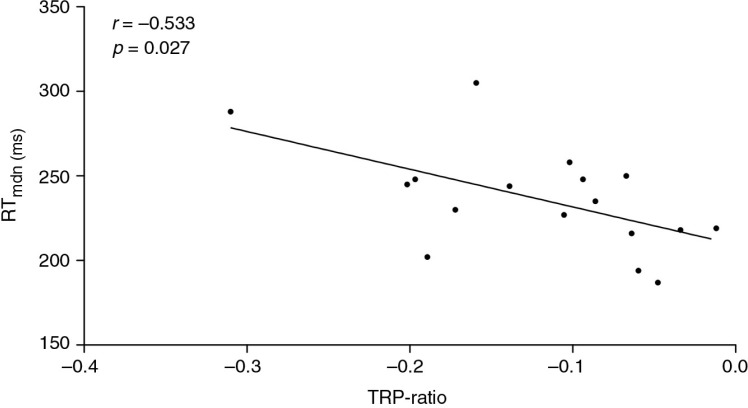
Correlation between plasma TRP-ratio and the median reaction time (milliseconds) in the fourth run of the PTD-challenge group (*p*=0.027, *r*=−0.533).

A dimensional analysis indicated a significant positive correlation between the reaction times and the DA-related depletion magnitude. The lower the TYR concentration was (which equaled a stronger depletion magnitude), the slower reaction times for the first run of the task were for the participants who received the PTD challenge (*r=*0.534, *p*=0.027, Fig. 2); the Cohen's *d* value of ≥1 indicated a medium to large estimated effect (41). For the remaining runs, no significant associations between the TYR concentrations and reaction times were identified in the same group. A higher TRP concentration in the same challenge group (PTD) was associated with a slower reaction time in the fourth run of the task, which also had a medium to large estimated effect size (*r*=−0.533, *p*=0.027, Cohen's *d*≥1, Fig. 3). However, the remaining runs exhibited no significant relationships between the TRP concentrations and the reaction times in the PTD group.

Regarding statistical power, based on the data obtained in the present investigation, to detect an average significant difference in means (*t*-values for phasic alertness) of 0.089 between the ATD and BAL groups with an alpha-level of 0.05 (two-tailed, unpaired *t*-test), a power of 95% was calculated. However, the difference identified in the present sample was 0.020. Regarding the comparison of the PTD and BAL groups, to detect an average significant difference in the means (*t*-values for phasic alertness) of 0.085 between the PTD and BAL groups with an alpha-level of 0.05 (two-tailed, unpaired *t*-test), a power of 95% was calculated; however, the difference observed was 0.04. Thus, future studies must include substantially larger samples. This pilot study provides an estimate for the calculation of sample sizes for future large-scale research.

## Discussion

In the present study, no significant group differences were identified. However, a dimensional analysis indicated that in the PTD challenge group, lower TYR concentrations as a surrogate for decreased DA-related precursor availability were correlated with slower reaction times in the first run of the task. In contrast, a higher TRP concentration in the same group was correlated with a slower reaction time in the fourth run of the task, whereas other runs of the task did not exhibit this association. Overall, the study findings are largely negative, which could be because of the sample size, as supported by the data obtained from the post-hoc power analysis. However, the lack of differences in alertness performance between the three challenge groups could also be because of functional and intact compensation mechanisms. This concept appears plausible from biological and, in part, genetic viewpoints because none of the subjects had a personal or family history of a psychiatric disorder, which has been shown to influence mood, motivational processes and neural reward pathways after monoamine depletion (42–45). Moreover, phasic alertness is one of the most basic aspects of human attention. A basic requirement of such important and basic functions is stability against external effects. Changes in the plasma TRP ratio occurring after a carbohydrate-rich meal may slightly modify 5-HT synthesis. However, an increase in circulating TRP in the periphery cannot be equated to equivalent changes in brain TRP concentrations, in particular because brain TRP availability depends on the ratio of TRP to LNAAs. Factors possibly influencing TRP supply are therefore frequency, size, and composition of meals ingested and the resultant insulin secretion (of note, no subject had diabetes, and all subjects participated after an overnight protein fast and also had a standardized breakfast, which is a standard procedure in ATD-related research).

Attentional performance is somewhat more vulnerable to dietary depletion of DA, whereas 5-HT is more responsible for impulsive behavior (46). There is evidence that the ascending serotonergic system has inhibitory effects on other monoamine and cholinergic neurotransmitter systems, in particular, regarding behavioral inhibition and cortical de-arousal (30). Similar to DA, 5-HT plays an important role in many physiological functions. Hence, 5-HT depletion may improve attentional control in the short term. This particular finding could explain the identified correlation between TRP and RT_mdn_ in the PTD group, with the elevated TRP concentrations serving as a potential cause of the slower reaction times. However, higher TRP concentrations cannot be the sole explanation for the slower reaction times identified because they cannot explain the negative findings in the balanced group with similar TRP concentrations after AA ingestion. These observations could be explained through a declined SNR in the participants of the PTD-challenge group. In particular, DA optimizes signal processing via the augmentation of the SNR (23, 24). Mehta et al. demonstrated that MPH may have improved response to signals with concomitant suppression of background noise and thereby improved the task results of participants, which depended on the individual baseline levels in the task (47). Although DA and 5-HT have distinct and even partially opposed functions regarding attentional control, both systems significantly interact because their functions in prefrontal cortical networks are inextricably related (48). The interactions between these two neurotransmitter systems have been emphasized in several other studies. Harrison demonstrated that increased impulsiveness in 5-HT-depleted rats, which was caused by brain lesions, was blocked through the administration of a D1-receptor antagonist (49). Regarding the detected preliminary associations, if these findings are replicated in larger studies, the findings of the present study together with previous research may suggest that short-term decreases in the synthesis rates of central nervous system DA and 5-HT, which are achieved by dietary depletion strategies, lead to dysfunctional interactions between these two ascending neurochemical systems.

The dietary depletion challenge administration (ATD, PTD) resulted in significantly lowered TRP, PHE, and TYR concentrations and TRP, PHE, and TYR ratios, respectively. ATD decreased the TRP ratio by 65%, whereas PTD lowered the PHE ratio by 65% and the TYR ratio by 82%. Overall, these data indicate that the neurochemical depletion paradigms had the intended effect. Recent studies in mice that used the same Moja-De AA-mixtures as humans confirmed the reduced brain 5-HT and DA synthesis after challenge administration (1, 35). Moreover, indirect evidence originates from studies in humans that used plasma concentrations of relevant AAs (3, 7, 10, 29, 34), the prolactin response to fenfluramine administration (50) or corresponding metabolites (9) as markers for successful depletion. It must be mentioned that in the past, multiple and quite diverse dietary depletion formulas have been used to alter serotonergic and dopaminergic neurotransmission, which complicates a detailed comparison (3, 5, 6, 8, 10, 28, 29, 34, 51). Furthermore, PTD-treated mice exhibited changes in brain TRP in the frontal cortex compared with the BAL condition (35). These results are of particular importance specifically because prefrontal cortical functions play a major role in attentional control, which is mediated through the ascending dopaminergic and serotoninergic projections (46).

Deficiencies in attentional control, motivational processes, memory, and behavioral processes are present in different clinical populations, such as in patients with ADHD. The present study is the first study to identify preliminary data that indicates lower plasma TYR levels after PTD were correlated with slower reaction times in specific runs of the task used. A decrease in reaction time tasks has been demonstrated in previous research using dietary depletion of TYR and PHE to attenuate central nervous system DA function, which suggests a role for DA in motor readiness and other cognitive functions (30). Further work has identified baseline-dependent improvements in cognitive function after the administration of D1 receptor agonists in rats (with individual differences in performance) (52). With regard to a review of the effects of ATD administration on memory, attention, and executive functions, our findings are somewhat conflicting because no effect of dietary ATD administration on sustained or divided attention was identified (27). In contrast, it was shown that involuntary attention shifting was altered after ATD administration, which was indexed by effects on EEG data (53).

Increased TRP influx across blood–brain barrier can be observed after both BAL and PTD challenges (3, 35). An effective TRP-loading can be achieved with doses of TRP in relation to the competing AAs and an even lower dose than used in this study (54–56). Overall, if confirmed by future larger studies, the preliminary results from the dimensional analysis could suggest that in subjects with an altered DA synthesis, TRP loading may positively affect attentional control. However, at this stage, this finding remains speculative and must be further investigated.

Some advantages of this study should be highlighted. The dietary depletion method (Moja-De) used is advantageous because of its acceptable tolerability, which enables its use even in children and adolescents (12, 29, 34, 57). The body weight–adapted dosing is likely to reduce side effects without affecting the depletion magnitude, which has been demonstrated in rodents and humans. As expected, depletion resulted in significantly lowered TRP, PHE, and TYR concentrations.

The present study also has several limitations. The limited sample size and the between-subjects design must be considered confounders. Moreover, the challenge conditions (ATD and PTD) used are thought to affect the whole brain, which raises the likely problem of regional differences in ATD and PTD induced changes in central nervous system neurotransmitter synthesis. Using the outlined depletion paradigms, it is not possible to elucidate specific dopaminergic or serotonergic functions related to different brain regions and isolated neurocircuits. Depletion may vary from one brain region to another region (58, 59). Studies that combine functional neuroimaging techniques and depletion and other neurochemical concepts are of particular value to examine the impact of different neurotransmitters in healthy and vulnerable populations regarding different brain regions (58, 59).

To our knowledge, this is the first study to investigate the effects of dietary ATD and PTD administration on phasic alertness in healthy adult volunteers. Overall, the results were primarily negative. However, if confirmed by future research, the results of the conducted dimensional analysis of the present pilot study may support the complex nature of serotonergic and dopaminergic interactions in the regulation of phasic alertness, which may have implications for the better understanding, and consequently, treatment of neuropsychiatric disorders related to monoaminergic dysfunctions. Future research with larger samples is necessary to further disentangle the relationship between the central nervous system availability of 5-HT and DA, with the potential use of dietary depletion protocols that aim to alter neurotransmitter synthesis with regard to related dietary precursor availability.
